# Magnetic resonance myocardial T1ρ mapping: Technical overview, challenges, emerging developments, and clinical applications

**DOI:** 10.1186/s12968-023-00940-1

**Published:** 2023-06-19

**Authors:** Aurelien Bustin, Walter R. T. Witschey, Ruud B. van Heeswijk, Hubert Cochet, Matthias Stuber

**Affiliations:** 1 IHU LIRYC, Electrophysiology and Heart Modeling Institute, Université de Bordeaux, INSERM, Centre de Recherche Cardio-Thoracique de Bordeaux, U1045, Avenue du Haut Lévêque, 33604 Pessac, France; 2 Department of Cardiovascular Imaging, Hôpital Cardiologique du Haut-Lévêque, CHU de Bordeaux, Avenue de Magellan, 33604 Pessac, France; 3 Department of Diagnostic and Interventional Radiology, Lausanne University Hospital and University of Lausanne, Lausanne, Switzerland; 4 Department of Radiology, University of Pennsylvania, Philadelphia, PA USA; 5 Center for Biomedical Imaging (CIBM), Lausanne, Switzerland

**Keywords:** T1-rho, Cardiac, Mapping, Magnetic resonance imaging, CMR

## Abstract

The potential of cardiac magnetic resonance to improve cardiovascular care and patient management is considerable. Myocardial T1-rho (T1ρ) mapping, in particular, has emerged as a promising biomarker for quantifying myocardial injuries without exogenous contrast agents. Its potential as a contrast-agent-free (“needle-free”) and cost-effective diagnostic marker promises high impact both in terms of clinical outcomes and patient comfort. However, myocardial T1ρ mapping is still at a nascent stage of development and the evidence supporting its diagnostic performance and clinical effectiveness is scant, though likely to change with technological improvements. The present review aims at providing a primer on the essentials of myocardial T1ρ mapping, and to describe the current range of clinical applications of the technique to detect and quantify myocardial injuries. We also delineate the important limitations and challenges for clinical deployment, including the urgent need for standardization, the evaluation of bias, and the critical importance of clinical testing. We conclude by outlining technical developments to be expected in the future. If needle-free myocardial T1ρ mapping is shown to improve patient diagnosis and prognosis, and can be effectively integrated in cardiovascular practice, it will fulfill its potential as an essential component of a cardiac magnetic resonance examination.

## Background

A significant amount of modern cardiovascular magnetic resonance (CMR) research is the quest for new methodologies that would further improve contrast beyond established T1- and T2-weighted techniques [1]. In recent years, there has been growing interest in quantifying and identifying injured myocardial tissue with CMR and to improve its specificity to underlying biochemical composition and pathophysiology. A question then arises: can new CMR imaging technologies be developed to generate endogenous contrast based on the inherent physical properties of a specific myocardial tissue component?

At the clinical magnetic field strength of 1.5 T for example, the precession of ^1^H would occur at the Larmor frequency of approximately 64 MHz. However, to study biological processes such as the proton exchange between water and macromolecules, which occur at lower frequencies (in the order of 100 Hz to a few kHz), conventional T1- or T2-weighting imaging may not be sufficient. The low main magnetic field strength B_0_ that would be needed to resonate at these frequencies would be impractical for clinical imaging due to its inherently low signal-to-noise ratio (SNR).

In this context, T1-rho (known as “T1ρ” and pronounced “T-one-rho”) imaging provides a viable approach to study low-frequency processes without sacrificing SNR [2]. T1ρ is also called the spin–lattice relaxation time in the rotating frame, and it can be used to probe the slow molecular motional processes in the kHz range, which includes proteins such as collagen and amyloid. This indicates that T1ρ may be able to detect the presence of interstitial fibrosis and other myocardial disease processes based on large molecules directly, rather than indirectly through their effects on water, which is the case for T1 and T2 relaxation. As such, it provides additional information about tissues beyond conventional T1- and T2-weighted imaging. The application of the quantitative form of T1ρ-weighted imaging (T1ρ mapping) to disc degeneration, articular cartilage, liver fibrosis, Alzheimer’s and Parkinson’s disease, stroke, and tumors is constantly progressing, and clinical applications appear to be maturing [3].

Evidence is also accumulating that endogenous T1ρ mapping of the myocardium can provide important molecular information about diseased myocardial tissue [4]. In fact, the non-invasive and contrast-agent-free quantification of myocardial fibrosis may become very attractive for the detection and characterization of heart disease. The method could allow fibrosis assessment in patients with kidney failure, who are currently unable to receive certain gadolinium-based contrast agents (GBCAs) due to poor kidney function. If proven as an accurate method for the assessment of myocardial fibrosis, T1ρ would not require administration of GBCAs, thus shortening the exam duration, lowering costs, and reducing risks. This in turn will simplify patient management while reducing MR operator workload and promoting serial CMR screening of patients for monitoring disease progression. More importantly, it may unlock the potential of CMR as a radiation-free method for the screening of children, pregnant women, and asymptomatic subjects at the population scale.

In this review, we aim at providing researchers and clinicians with an overview of the most-widely used myocardial T1ρ mapping tools from a more technical viewpoint with an emphasis on physical characteristics, sequence design, standardization, and influencing factors. We then discuss clinical applications and provide an insight to some of the emerging developments that may help bring myocardial T1ρ mapping closer to clinical practice.

## Physical basis of T1ρ

One of the first applications of T1ρ to magnetic resonance imaging (MRI) was investigated by Sepponen et al. [5] nearly 40 years ago, but the “spin-locking” concept is far older and embraces many elements developed in the mid 1950s [6]. While T1 and T2 relaxation times are essentially physical properties of tissue at a given magnetic field strength, T1ρ is unique in that the relaxation time depends on the properties of tissue as well as on the applied spin-locking radiofrequency (RF) pulse and its features (amplitude, duration, and type of module). By varying these features, one can modulate the spin-locking pulse and investigate how water protons are affected by their environment (the “lattice”). Since the spin-locking fields are on the order of 2–12 μT (i.e., the low kHz range), T1ρ relaxation time is sensitive to slow molecular motion processes in the lattice and may therefore provide complementary information to conventional T1 and T2 measurements.

**Fig. 1:**
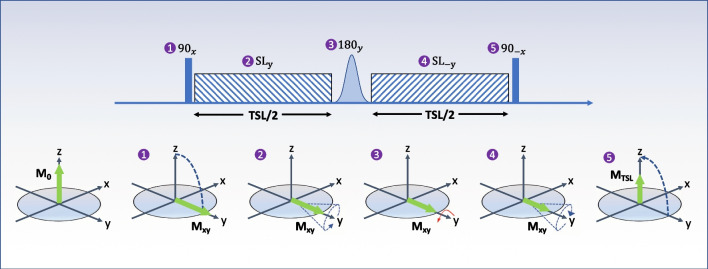
Illustration of a simple T1ρ spin-lock experiment. A first 90º “tip-down” radiofrequency (RF) pulse is applied along the x-axis (transverse plane) to rotate the magnetization, followed by two spin-lock pulses, with alternating phases (SL_±y_) and fixed duration, that are separated by an adiabatic 180º pulse. An additional 90º “tip-up” pulse is played out to flip back the magnetization to the z-axis (longitudinal direction). After the T1ρ preparation module, a crusher gradient is used to eliminate any residual magnetization in the transverse plane. *SL* spin-lock, *TSL* spin-lock time, *M*_*0*_ longitudinal magnetization, *Mxy* transverse magnetization

In a T1ρ MRI experiment, the equilibrium magnetization established by the static B_0_ magnetic field is rotated by 90º (tip-down) RF pulse into the transverse—M_xy_—plane. A spin-lock RF pulse with amplitude B_1_ is then applied parallel to the magnetization to lock the spins in the rotating frame. Then a tip-up 90º pulse is applied to flip the magnetization back to the longitudinal plane (Fig. 1). For T1ρ modules including one or more refocusing pulses (see “Sequence design” below), the T1ρ relaxation time transitions to the T2 (spin–spin) relaxation time as the spin lock amplitude, B_1_, approaches zero. T1ρ signal relaxation can be characterized by the following equation:

1 $$S\left(TSL\right)={M}_{0}{e}^{-\frac{TSL}{T1\rho }}$$

where S is the signal intensity, TSL is the spin-lock time, and M_0_ is the equilibrium magnetization. At clinical field strengths, spin-lock amplitudes are in the range of 100 to 500 Hz and TSL varies from 0 to 100 ms depending on the tissue of interest.

## What factors influence T1ρ?

Key mechanistic insights about T1ρ relaxation can be gleaned by its dispersion or the variation in T1ρ with the spin lock amplitude [7–12]. The T1ρ dispersion curve provides information about the biophysical mechanisms central to magnetic relaxation. Applied to articular cartilage imaging [13] for example, the R1ρ (= 1/T1ρ) dispersion curve was shown to consist of two distinct processes in the 0.1–6 kHz frequency range. In the low frequency range (range of spin-lock amplitudes employed on current MR systems), the relaxation rate increased as the tissue was degraded with trypsin and lost proteoglycan (which is an indicator of early osteoarthritis), whereas the high frequency component did not change significantly. As T1ρ dispersion is shown to increase during the evolution of the pathological process, it is an excellent contrast agent-free MRI marker for other conditions such as acute cerebral ischemia [14], liver fibrosis [15], or cancer [16].

Initial studies have investigated the sensitivity of T1ρ mapping to factors such as pH change, oxygen saturation, diffusion, and collagen content. Each of these components is discussed in more detail below.

### Sensitivity to macromolecule–water interactions and collagen content

In biological tissues with proteins, proton exchange is expected to contribute to T1ρ relaxation. Several groups have explored the relationship and positive correlation between T1ρ and tissue water ^1^H content [17, 18]. Mäkelä et al. have shown that proton exchange serves as a relaxation mechanism for T1ρ at spin-lock frequencies ranging from 1 to 11 kHz (range used for in vivo experiments), and that it is the dominant mechanism for the R1ρ dispersion [17]. In the 0.1–10 kHz spin-lock range, T1ρ relaxation and dispersion have been shown to be sensitive to macromolecule-water interactions in various protein solutions, with R1ρ increasing concomitantly with molecular concentration and weight [19, 20]. On the one hand, T1ρ is sensitive to interactions between tissue water and the macromolecular environment on the time scale of the spin-locking field. On the other hand, increased myocardial fibrosis associated with extracellular matrix expansion may shorten water ^1^H rotational correlation times (i.e., the average time it takes for a molecule to rotate one radian) [21]. One can thus expect an increase in T1ρ values for a wide range of myocardial injuries associated with extracellular matrix expansion.

Regarding collagen content, Zhao et al. [22] found a strong and significant positive correlation (R = 0.82, slope of 1.35) between liver collagen content and liver T1ρ in rats with non-alcoholic fatty liver disease. Since collagen content is a feature of many cardiomyopathies, ranging from scar formation to cardiac remodeling, its detection with endogenous T1ρ relaxation could be of significant clinical value.

### Sensitivity to pH change and chemical exchange

There have been few studies undertaken that have shown the contributions of chemical exchange to T1ρ dispersion in tissue models [23, 24]. The two most used MRI techniques sensitive to proton exchange are chemical exchange saturation transfer (CEST) and spin-lock MRI [16, 25, 26]. Jin et al. found that chemical exchange-sensitive spin-lock MRI was more sensitive than CEST at 9.4 T and would be particularly valuable for amine- or hydroxyl-water proton exchange studies [25, 27]. Using adjusted adiabatic spin-locking at this field strength was shown to provide higher chemical exchange weighting than conventional CEST [28]. Several studies have reported a significant sensitivity of R1ρ measurements to changes in pH with higher T1ρ relaxation times corresponding to a lower pH (i.e., more acidic) [29–31]. A similar trend was observed using spin-lock amplitudes within FDA safety guidelines (i.e., less than 1000 Hz) [32]. In brain imaging, Kettunen et al. observed a pH-dependent decreased in R1ρ measurements in the ischemic rat brain [33]. In a different brain study, T1ρ also correlated closely with pH measurements [30]. Since pH regulation is an important factor contributing to electrical disturbances and to myocardial injury associated with ischemia–reperfusion of the heart through a variety of mechanisms [34–36], the non-invasive imaging of pH using endogenous T1ρ could have important implications in myocardial ischemia imaging.

### Sensitivity to oxygen saturation and diffusion

Using phantoms at 4.7-T, Kettunen et al. showed that T1ρ of blood was linearly related to oxygen saturation [31], which allows the effects of activation-induced changes in blood volume and saturation to be measured. Blood oxygen saturation dependence of T1ρ was observed in brain imaging, with T1ρ of blood increasing with increasing hemoglobin oxygen saturation [31, 37]. Spin-locking pulses can be added to functional MRI sequences to detect blood oxygenation level-dependent (BOLD) signals and emphasize small vascular structures in the brain [38].

Diffusion effects also affect the rate of spin–lattice relaxation in the rotating frame. This contribution can be mitigated by increasing the spin-locking field. Spear et al. [39, 40] derived an analytic relationship between the rate of diffusion within a sinusoidally spatially varying gradient field and the dispersion of R1ρ. This theoretical expression can be employed to quantify diffusion effects for R1ρ. A potential clinical application is the enhanced localization of local changes in blood oxygenation level resulting from neural activation.

### Evidence for these mechanisms in in vivo myocardium

Data that links the previous findings to the in vivo heart are limited. Witschey et al. [21] found that there exist MR relaxation mechanisms that operate below a spin-lock field strength of 500 Hz at 3.0 T and which suppress endogenous contrast between mature scar, myocardial tissues, and healthy myocytes in a swine model of infarction. However, by delivering a spin-lock pulse above 500 Hz, they found that it becomes possible to overcome these mechanisms and to reveal strongly elevated differences in relaxation rate between tissue types. On a 7 T system, differences in T1ρ increased between remote myocardium, infarct, and border zone upon increasing the spin-lock amplitude from 0 to 2500 Hz (∆T1ρ_Infarct/Remote_ = 74 ms at 500 Hz vs. ∆T1ρ_Infarct/Remote_ = 137 ms at 2500 Hz) [21]. A fast (fourfold) technique for T1ρ dispersion imaging of the heart in mice was also proposed by Gram et al. [41].

**Fig. 2:**
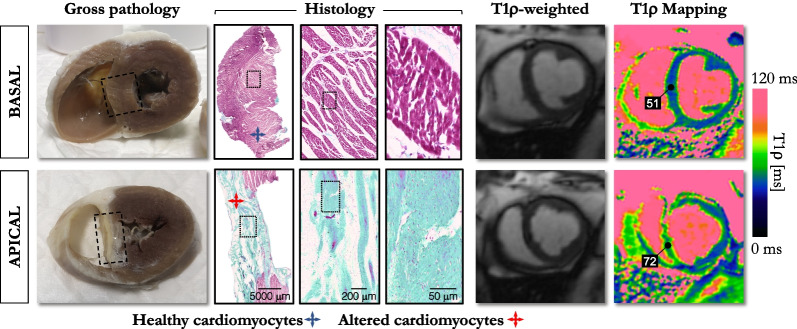
Visual correspondence between histology and T1ρ maps in a pig with induced myocardial infarction. The chronic model was developed by percutaneous coronary artery embolization and T1ρ mapping was carried out 3 months post myocardial infarction on a 1.5-T system using a T1ρ -prepared sequence. Slices were then stained with Masson’s trichrome to visualize healthy cardiomyocytes (red) and collagen (green). Transmural infarction covering the whole septum can be observed in gross pathology, histology, and T1ρ maps. Septal T1ρ elevation (72 ms vs. 51 ms) corresponded with the collagenous zone identified on histological data

To further investigate the evolution of T1ρ relaxation times for different myocardial tissue compositions, we correlated histology data and T1ρ obtained in a pig model of myocardial infarction on a 1.5-T system, three months after the infarction was induced. The study found that T1ρ elevation (72 ms vs. 51 ms) in the apical septal region corresponded well with the collagenous zone identified on histological data (Fig. 2) [42].

## Myocardial T1ρ mapping: sequence, reconstruction, analysis

### Sequence design

**Fig. 3:**
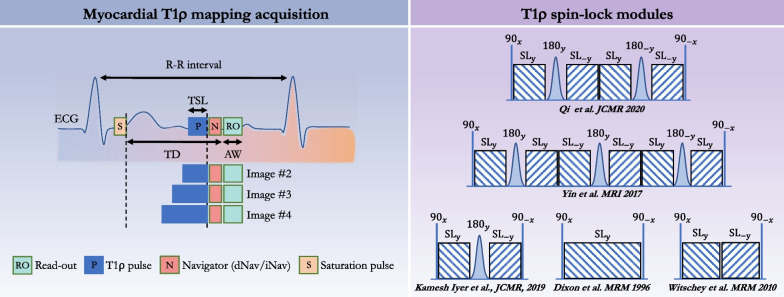
Schematic overview of a myocardial T1ρ mapping acquisition framework (left) and existing T1ρ-preparation modules (right). Multiple T1ρ weighted images are collected with electrocardiogram (ECG) triggering used to minimize cardiac motion by synchronizing the data acquisition or readout (RO) with the heart motion. For 3-dimensional free-breathing applications, a navigator (diaphragmatic-based [dNav] or 2-dimensional image-based [iNav]) is integrated before data acquisition to compensate for respiratory motion. A nonselective saturation pulse (S) can also be added after each R wave to effectively null the longitudinal magnetization, to reduce the sensitivity to RR variability, and avoid the use of recovery periods. Right side: T1ρ spin lock modules with and without adiabatic and refocusing pulses. *AW* acquisition window, *SL*_*±y*_ spin-lock direction, *TD* trigger delay, *TSL* spin-lock time

**Fig. 4:**
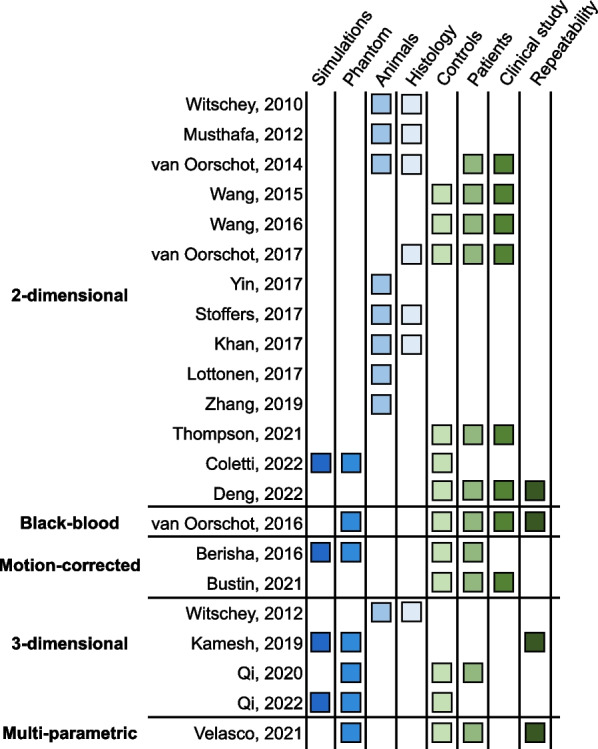
Chart of studies and experiments performed using myocardial T1ρ mapping

A general cardiac acquisition scheme is illustrated in Fig. 3. An electrocardiogram (ECG)-triggered pulse sequence is used to acquire multiple images at different spin-lock times along the T1ρ decay curve. In practice, the trigger delay is adapted to ensure that the images are acquired in the same quiescent cardiac phase (usually mid-diastole). Two heartbeats typically separate each acquisition to allow for enough magnetization recovery in between spin-lock cycles, although this can be adjusted to the patient’s heart rate. In 2-dimensional (2D) imaging, three short-axis slices (basal, mid-cavity, apical) are commonly collected, each of them during one breath-hold. Most studies have used between five and eight T1ρ-weighted images to generate the quantitative T1ρ map. A variety of T1ρ composite pulse clusters have been reported in the literature, each with specific advantages and limitations, and are shown in Fig. 3. A commonly used pattern is the 2-spin-lock scheme (also called a rotary echo) separated by a 180º adiabatic refocusing pulse. It should be noted that the adiabatic refocusing pulses [43] also use spin-locking, and that their T1ρ locking frequency varies due to their fluctuating amplitude [44]. Moreover, the radiofrequency (RF) power deposition of adiabatic pulses can sometimes challenge SAR limits in vivo, which is exacerbated at higher field strength. To satisfy the adiabatic condition with reduced RF power deposition, a stationary spin-locking field can be applied using a train of amplitude- and frequency-modulated pulses operating in a sub-adiabatic condition (RAFF [45, 46]). Typical imaging parameters for myocardial T1ρ mapping at 1.5-T are provided in Table 1. An overview of several myocardial T1ρ mapping techniques and their validation approaches is shown in Fig. 4.

**Table 1:** Standard pulse sequence parameters and protocol setting for magnetic resonance myocardial T1ρ mapping at 1.5-T

Sequence setting	Parameter range	Notes
Acquisition	(2D) Single-shot SSFP (3D) SPGR	Allows for rapid 2D image acquisition that is robust to motion, especially in arrhythmic patients or poor breath-holders
Cardiac control	ECG triggering	Usually performed in mid-diastole to minimize the effects of cardiac motion
Respiratory control	(2D) Breath-holding (3D) Free-breathing navigated	Non-rigid motion correction is recommended even under breath-holding [42, 59, 80]
Spatial resolution	1.4 × 1.4 mm^2^ to 1.7 × 1.7 mm^2^	Balance the image resolution and the amount of expected cardiac motion
Slice thickness	8–10 mm	Can be reduced for 3D acquisitions
Acquisition window	160–250 ms	For 3D imaging, increasing the data acquisition window will reduce scan time but will increase cardiac motion
Bandwidth	900 Hz/pixel	–
Flip angle	70º	Single-shot imaging with a flip angle of 70º was shown to achieve low mean T1ρ bias in phantom experiments [59]
Recovery heartbeats	3	To allow for sufficient T1 recovery when no saturation pulses are incorporated in the sequence. This could also be countered with dictionary matching
Acceleration	(2D) GRAPPA R = 2 with 34 reference lines (3D) Variable density trajectory R = 3–4 [52, 55]	Increased acceleration (higher R) will reduce scan time (3D) or acquisition window duration but may also affect image quality. In 3D, advanced undersampling and reconstruction strategies may allow high acceleration (R = 3–4) [52, 55]
T1ρ number	5–7 (durations = 0 to 55 ms)	The optimal number of pulses and corresponding spin-lock durations still have to be optimized (resorting to Cramér-Rao Lower Bound for example [72])
T1ρ durations	0, 10, 20, 35, 50 ms	
Spin-lock frequency	400 Hz (B1 amplitude: 9.4 μT)–500 Hz (11.7 μT)	To stay within the allowed specific absorption rate limits
T1ρ module	${90}_{x}-S{L}_{-y}-{180}_{-y}-S{L}_{-y}-{90}_{-x}$	This module inserts a 180º refocusing pulse between the two spin-lock segments and was shown to be insensitive to B0 and B1 inhomogeneities [3]. A thorough analysis of all existing T1ρ modules remains to be performed
Acquisition time	Spin-lock number $\times$ Recovery heartbeats	In practice, single-shot 2D acquisitions require a breath-hold per slice. Depending on the resolution, the motion correction technique, and the sampling acceleration strategy, 3D T1ρ maps can be acquired in less than 10 min

*GRAPPA* generalized autocalibrating partially parallel acquisitions, *TE* echo time, *TR* repetition time, *RR* time interval between two consecutive R waves, *SSFP* steady-state free-precession readout, *SPGR* spoiled gradient echo, *TSL* spin-lock time

**Fig. 5:**
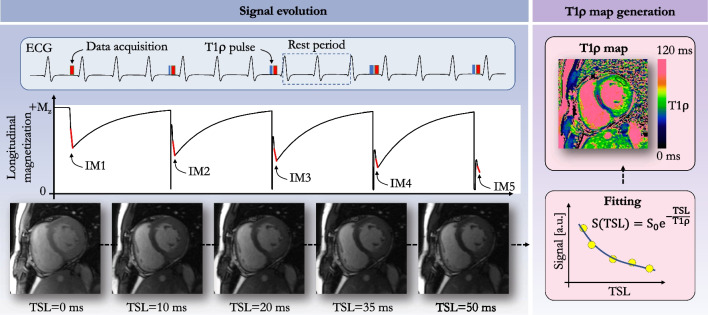
T1ρ magnetization signal evolution (left) and data fitting (right). *ECG* electrocardiogram, *TSL* spin-lock time

A limitation of the breath-held 2D T1ρ mapping sequence is the need to acquire the data over multiple heartbeats (Fig. 5) in patients who have difficulty holding their breath. In reality, and congruent with other mapping techniques, even patients who can hold their breath often do not adhere to breathing instructions from the MR technician. Indeed, residual respiratory drift of the heart is often observed during a breath-hold (an average displacement of 5.1 ± 2.7 mm was reported in a recent study including 30 adult patients [42]) while inconsistencies of the diaphragmatic position among serial breath-holds and fluctuating R-R intervals may have to be considered as well [47–49]. It is therefore strongly recommended to use motion correction strategies to improve the robustness and clinical acceptance of myocardial T1ρ mapping.

Another issue is that if insufficient time is allowed for magnetization recovery, the measured T1ρ values would become heartrate dependent, SNR of T1ρ-weighted images would be reduced, and T1ρ fit quality would be compromised. A solution, borrowed from other mapping techniques [50, 51], has been proposed by Qi et al. [52] who added a nonselective saturation pulse after each R wave to effectively null the longitudinal magnetization at each heartbeat. Recovery heartbeats between data acquisition are no longer needed, scan time is reduced, and systematic bias related to heart-rate dependence no longer present. The main drawback of using saturation pulses is that the magnetization may not fully recover, reducing SNR. Furthermore, as observed with late gadolinium enhancement (LGE) imaging, the bright-blood contrast of T1ρ-weighted images can hamper the robust detection of subendocardial lesions adjacent to the blood pool. To overcome this issue, Van Oorschot et al. [53] harnessed the potential of black-blood imaging for myocardial T1ρ mapping. In that study, the authors made use of a double inversion preparation pulse (combining nonselective and selective 180º pulses) to generate a black-blood contrast. Compared with LGE, they showed a sensitivity of infarct detection of 69% and a specificity of 94% using black-blood T1ρ mapping.

### Myocardial T1ρ map reconstruction and data fitting

**Fig. 6:**
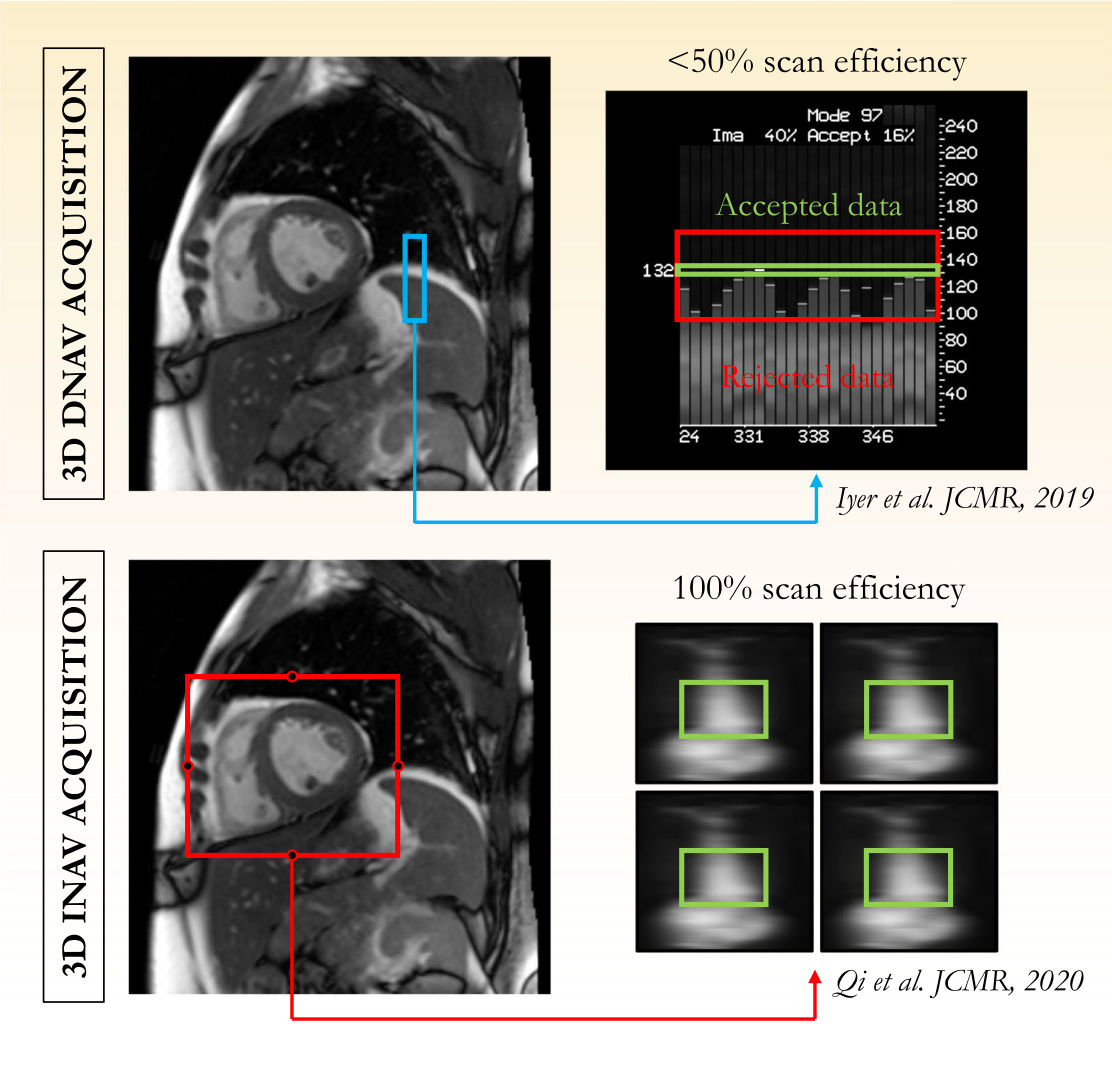
Motion correction strategies available for Cartesian 3-dimensional T1ρ mapping of the heart. Free-breathing 3-dimensional myocardial T1ρ mapping can be performed by using diaphragmatic navigation as proposed by Iyer et al. [55] or using image-based navigation as proposed by Qi et al. [52]. While both techniques enable motion corrected T1ρ mapping of the heart, the use of iNavs allows for 100% scan efficiency (i.e., no data rejection) which ultimately leads to faster scan times

Two-dimensional T1ρ-weighted images are usually reconstructed with a conventional generalized autocalibrating partially parallel acquisitions (GRAPPA) reconstruction, which is widely available on commercial systems [54]. Typical acceleration factors of 2 to 3 may shorten the acquisition times and reduce the influence of cardiac motion when single-shot imaging is used. For free-breathing 3-dimensional (3D) applications [52, 55], two T1ρ mapping sequences have been proposed. Both techniques employ a Cartesian variable-density k-space trajectory to reach high acceleration factors (3- and fourfold) but differ in the way respiratory motion of the heart is handled (Fig. 6) and how images are reconstructed. The first technique, proposed by Iyer et al. in 2019, uses a compressed-sensing framework independently on each T1ρ-weighted image to reconstruct high-quality—denoised—volumes. The motion correction is handled during data acquisition with a diaphragmatic navigator (mean scan efficiency of 54.5% ± 12, total scan time of 18 min at 1.9 × 1.9 × 6 mm^3^ spatial resolution). The second approach, proposed by Qi et al. in 2020 [52], combines image-based navigation with a high-order patch-based reconstruction which, as opposed to the previous techniques, exploits the significant redundancy observed in the T1ρ-weighting dimension (scan efficiency of 100%, total scan time of 6 min at 1.7 × 1.7 × 2 mm^3^ spatial resolution).

Once the images have been reconstructed, the fitting procedure should be carefully considered. In most studies, T1ρ maps are computed on a pixel-by-pixel basis using the monoexponential decay model defined in Eq. (1), similar to T2 mapping. A linear fit can be employed after log transformation of Eq. (1):

2 $$\mathrm{ln}\left(S\left(TSL\right)\right)=\mathrm{ln}\left({M}_{0}\right)-\frac{TSL}{T1\rho }$$

Equation (2) can be written as $y=Ax+b$, with $y$ being $\mathrm{ln}\left(S\left(TSL\right)\right)$, $x$ being $TSL$, the slope $A$ being $-\frac{1}{T1\rho }$, and the intercept $b$ being $\mathrm{ln}\left({M}_{0}\right)$. $A$ and thus T1ρ can be obtained by linear fitting given different T1ρ-weighting images ($y$) acquired at different spin-lock times ($x$). A three-parameter non-linear least square fit using the Levenberg–Marquardt algorithm can also be considered [53, 56] and can help to address limitations observed with the two-parameter model. These include the possibility to fit short T1ρ times and T1ρ-weighted images with low SNR.

### Image analysis and T1ρ map quality assessment

**Fig. 7:**
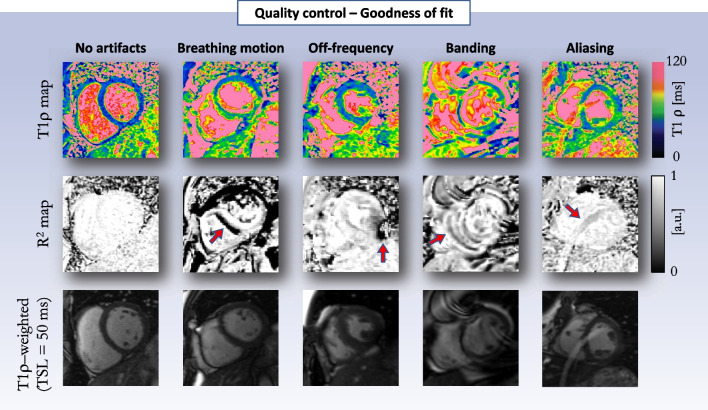
Examples of artifacts observed on T1ρ maps and corresponding “goodness-of-fit” R^2^ maps. For optimal diagnostic confidence, clinicians should review the source T1ρ-weighted images, T1ρ maps, and corresponding R^2^ maps during scanning. The red arrows indicate artifacts

As with any cardiac mapping sequence, the quality of the source T1ρ-weighted images and reconstructed T1ρ maps should be inspected. Quality metrics such as R^2^ and adjusted R^2^ (prediction-based fit) or root mean square error (uncertainty) maps should be included in the interpretation and evaluated during scanning to allow for repeated acquisitions if quality is deemed insufficient. R^2^ maps are calculated on a pixel-by-pixel basis and are dependent on a wide range of factors including respiratory-related motion, off-resonance artifacts, slice profiles, dark banding-like artifacts, or aliasing interference. Figure 7 shows several examples of artifacts observed on myocardial T1ρ mapping experiments with the corresponding R^2^ maps. Myocardial segments with low R^2^ values (i.e., below R^2^ < 0.90 [57]) must be excluded during analysis.

## Sources of variability, counfounding factors, and considerations

**Fig. 8:**
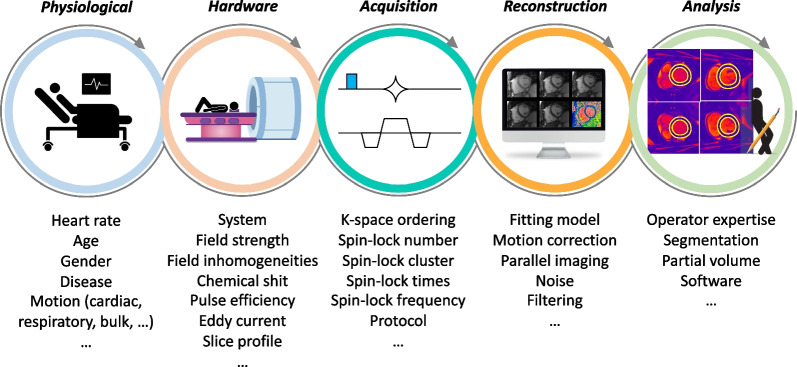
Identified factors potentially impacting T1ρ values reproducibility and variability. Adapted with permission from Ogier et al. [58]

Any reported relaxation time is the result of the combination of the subject, hardware, acquisition, reconstruction and fitting algorithms, and map analysis that are being used [58]; consequently, all steps in obtaining a T1ρ relaxation time can add bias or uncertainty to its measurement (Fig. 8). In this section, we will only briefly discuss the main factors potentially impacting T1ρ values reproducibility and variability and we refer the reader to [59] for a more detailed discussion.

*On the physiological side* several factors can influence myocardial T1ρ values. Gender, age, heart rate, temperature, body mass index and disease are important factors to consider [60–62]. Multiples studies have already reported higher relaxation times (e.g., T1 and T2) in females. But the true effects of gender and age remain unclear for myocardial T1ρ mapping. More investigations must be undertaken to better understand the impact of physiological factors that drive T1ρ changes.

*On the hardware and acquisition sides*, and besides obvious magnetic field strength dependence, spin-lock pulse efficiency must be considered. Compared to standard Malcolm Levitt (MLEV) schemes [63], self-compensating and adiabatic pulses have been proposed to compensate for B0 and B1^+^ inhomogeneities [64, 65]. These schemes reduce artifacts caused by spin-lock pulses with constant amplitude [66–71], but the compound spin-locking frequency of such adiabatic RF pulses has not been well-characterized. The optimal T1ρ scheme to employ for myocardial T1ρ mapping as well as its duration and the optimal number of points to sample is subject of ongoing research. To this end, resorting to the Cramér-Rao Lower Bound framework may be particularly useful [72].

Giri et al. [73] have also observed image artifacts in subjects when using centric k-space ordering for myocardial T2 mapping (vs. linear ordering) which is likely caused by oscillatory approach to steady-state. This would have to be assessed for myocardial T1ρ mapping as well [59].

*On the reconstruction and analysis sides*, the appropriate choice of the fitting model (2-point, 3-point, dictionary matching) is of utmost importance as it may impact the accuracy and precision of myocardial T1ρ values [59, 72]. Besides the fitting model, the sensitivity of T1ρ mapping to detect myocardial injuries is dependent on the number and timing of the spin-lock pulses, the image SNR, and the tissue of interest. As developed for myocardial T1 mapping, a calibration map that could quantify the quality of T1ρ estimate would be clinically valuable [74]. Finally, and as with other CMR mapping techniques, T1ρ map analysis requires manual segmentation of endocardial and epicardial contours, which is prone to human errors. Several potential avenues are discussed in the next section.

*Considerations.* A number of published articles have raised specific absorption rate (SAR) concerns related to spin-locking pulses [3, 75]. RF power deposition has indeed been one of the challenges preventing myocardial T1ρ mapping to be widely used in clinical practice as spin-locking pulses may approach FDA-specified SAR limitations. However, there have been solutions to mitigate this issue including reducing spin-locking frequency (0 to 500 Hz) or using off-resonance pulses [76, 77]. Partial k-space sampling, in which a full power spin-lock pulse is used for the central phase-encode lines of k-space while the remainder lines receive a low-power spin-lock pulse, can also be investigated [75].

## “Normal” T1ρ values

Publications reporting normal healthy values of the left ventricle obtained across different system vendors, field strengths, pulse sequences, and imaging sites are listed in Table 2. While multiple studies report normal T1ρ values in the order of 50 ms at 1.5 T and 3.0 T, significant variability exists among centers and sequences. This observation calls for the standardization of acquisition and interpretation methods for myocardial T1ρ mapping. The protocols that define the conditions in which T1ρ maps are acquired and the ways the data are reconstructed and fitted should be standardized. Repeated measurements in standardized phantoms (e.g., NIST [78] or T1MES [79]) and healthy volunteers should also be performed to determine stable cut-off values for the differentiation of healthy and diseased tissue.

**Table 2:** Reported sequences and normal myocardial T1ρ values in healthy subjects according to sequences and field strengths

Study	Year	Magnet	System	Sequence	Spin-lock module			Number of subjects*	Age (y)	Normal T1ρ (ms)
					Scheme	#TSL	Amplitude			
van Oorschot J et al. [83]	2014	3.0-T	Achieva, Philips	2D SSFP	Cluster 1	4	750 Hz	5 (0)	25 ± 3	50.0 ± 3.0
Wang C et al. [81]	2015	3.0-T	Trio, Siemens	2D GRE	Cluster 1	3	341 Hz	8 (5)	43 ± 10	42.2 ± 11.6
Wang L et al. [57]	2016	3.0-T	Verio, Siemens	2D GRE	NR	5	400 Hz	35 (20)	41 ± 14	49.4 ± 22.6
Berisha S et al. [59]	2016	1.5-T	Avanto, Siemens	2D SSFP	Cluster 1	7	500 Hz	10 (3)	29 ± 7	64.5 ± 2.1
van Oorschot J et al. [53]	2016	1.5-T	Ingenia, Philips	2D SSFP	Cluster 1	5	500 Hz	10 (2)	27 ± 3	52.8 ± 1.8
van Oorschot J et al. [53]	2016	3.0-T	Achieva, Philips	2D SSFP	Cluster 1	5	500 Hz	10 (6)	30 ± 3	46.4 ± 1.8
van Oorschot J et al. [56]	2017	3.0-T	Achieva, Philips	3D GRE	Cluster 1	5	500 Hz	8 (2)	51 ± 6	51.5 ± 1.2
Kamesh Iyer S et al. [55]	2019	1.5-T	Avanto, Siemens	3D SSFP	Cluster 1	12	500 Hz	6 (NR)	NR	67.9 ± 4.5
Qi H et al. (53)	2020	1.5-T	Aera, Siemens	3D SSFP	Cluster 2	5	400 Hz	11 (5)	30 ± 3	58.0 ± 4.1
Bustin A et al. [42]	2021	1.5-T	Aera, Siemens	2D SSFP	Cluster 2	5	500 Hz	8 (3)	30 (24–40)	47.7 ± 4.0
Thompson EW et al. [80]	2021	1.5-T	Avanto, Siemens	2D SSFP	Cluster 1	7	400–500 Hz	10 (6)	51 (38–55)	65.4 ± 5.2
Deng W et al. [107]	2022	1.5-T	Ingenia, Philips	2D SSFP	Cluster 1	4	400 Hz	57 (28)	27 ± 12	47.9 ± 2.8

Cluster 1: ${90}_{x}-S{L}_{-y}-{180}_{-y}-S{L}_{-y}-{90}_{-x}$. Cluster 2: ${90}_{x}-S{L}_{y}-{180}_{y}-S{L}_{-y}-S{L}_{y}-{180}_{-y}-S{L}_{-y}-{90}_{-x}$. *Number in parentheses indicates number of female subjects. GRE, gradient echo; NR, not reported; SSFP, steady-state free precession; TSL, spin-lock time

## Current clinical applications of myocardial T1ρ mapping

**Fig. 9:**
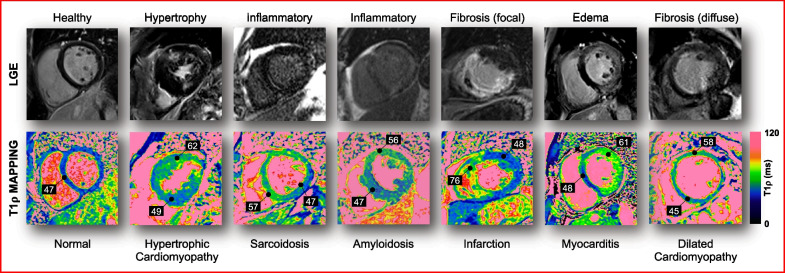
Application of contrast-agent-free myocardial T1ρ mapping in ischemic and non-ischemic cardiomyopathies. Images were acquired on a 1.5-T system (Magnetom Aera, Siemens) at Bordeaux University Hospital, France. *LGE* late gadolinium enhancement

To date, only ten studies have reported myocardial T1ρ values in patients, six of them being oriented towards specific populations (Table 3): hypertrophic cardiomyopathy (HCM) [80, 81], myocardial infarction [53, 82], idiopathic dilated cardiomyopathy (DCM) [56], end-stage renal disease [57], and mixed cardiomyopathy [42] (Fig. 9). We discuss these applications in the sub-sections below and refer the readers to Han et al. [4] for further reading.

**Table 3:** Myocardial T1ρ values and effect size under specific clinical conditions

Study population (n = Participants)	Healthy T1ρ value (ms)	Disease T1ρ values (ms)	Effect size (Cohen’s *d*)	References
Chronic myocardial infarction (n = 21)	54.0 ± 6.0	79.0 ± 11.0	2.8	Van Oorschot et al. [82]
Chronic myocardial infarction (n = 9)	52.8 ± 1.8	82.4 ± 5.2	7.6	Van Oorschot et al. [53]
Hypertrophic cardiomyopathy (n = 18)	42.5 ± 1.2	NR	NR	Wang C et al. [81]
Hypertrophic cardiomyopathy (n = 40)	65.4 ± 5.2	72.2 ± 5.8	1.2	Thompson et al. [80]
Dilated cardiomyopathy (n = 20)	51.5 ± 1.2	55.2 ± 2.7	1.5	Van Oorschot et al. [56]
End-stage renal disease (n = 32)	49.4 ± 22.6	52.2 ± 4.0	0.2	Wang L et al. [57]

*NR* not reported

### ST-elevation myocardial infarction

There are two studies reporting the use of myocardial T1ρ mapping in patients with chronic myocardial infarction. Both studies were performed on a 1.5 T system in patients with a first episode of reperfused ST segment-elevated myocardial infarction. In the first cohort of 9 patients [53], van Oorschot et al. reported significantly higher T1ρ values in the infarct area compared to remote tissue (82.4 ± 5.2 ms vs. 54.2 ± 2.8 ms, P < 0.0001). In the second cohort of 21 patients [82], the same authors also reported a similar result (79 ± 11 ms vs. 54 ± 6 ms, P < 0.0005) and found an agreement of 74% in segmental scar distribution (using the 17 segments AHA-model) measured on late gadolinium enhancement (LGE) images and T1ρ maps.

### Hypertrophic cardiomyopathy

**Fig. 10:**
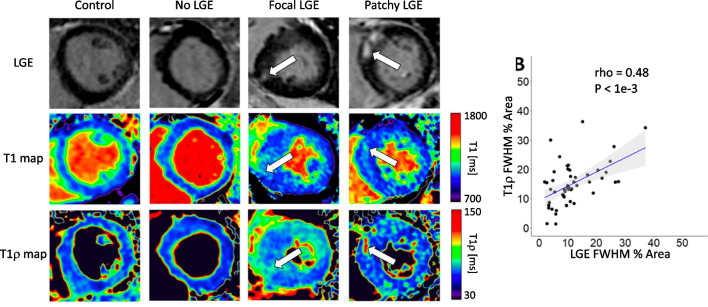
Native T1 and T1ρ maps and late gadolinium enhancement (LGE) images acquired at 1.5-T in a healthy control and in three patients with hypertrophic cardiomyopathy. *LGE* late gadolinium enhancement, *FWHM* full width half maximum. [80] Reproduced with permission from Thompson et al.

Hypertrophic cardiomyopathy (HCM) is a genetic cardiovascular disease characterized by an inappropriately increased left ventricular wall thickness in the absence of an obvious cause for the myocardial hypertrophy, such as systemic hypertension or aortic stenosis. The estimated prevalence of HCM is 1 in 500 people [83, 84]. LGE by CMR was shown to exhibit substantial prognostic value in sudden cardiac death events prediction in HCM patients [85]. There is also growing evidence regarding the predictive value of contrast enhanced CMR such as extracellular volume fraction (ECV) measurements and T1 mapping for ventricular arrhythmias and congestive heart failure in this population [86]. There have been continuous efforts to create more specific gadolinium-free CMR methods for myocardial fibrosis characterization in HCM patients. Wang et al. [81] investigated the use of T1ρ mapping for fibrotic assessment in 18 HCM patients at 3.0-T. They visually found elevated T1ρ values in 67% patients (values were not reported), which was consistent with LGE in terms of fibrotic sizes and locations. Furthermore, the extent of fibrosis determined using T1ρ mapping (through n-SD thresholding) correlated significantly with those determined by LGE. Thompson et al. [80] more recently sought to evaluate the role of contrast-agent-free myocardial 2D T1ρ mapping in 40 HCM patients compared to established native T1 and LGE at 1.5-T. The authors observed T1ρ value elevations in HCM patients (72.2 ms) compared with controls (65.4 ms, P = 0.618). The extent of native T1 and T1ρ abnormalities was also moderately correlated with the extent of LGE (Fig. 10).

### Dilated cardiomyopathy

**Fig. 11:**
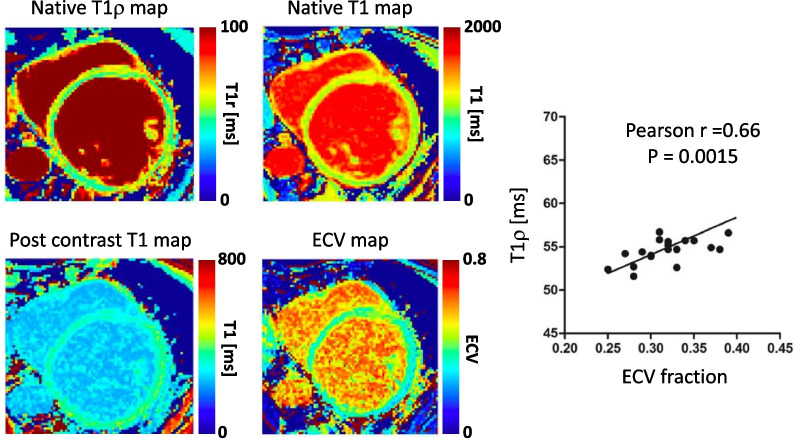
Myocardial native T1 and T1ρ maps and post-contrast T1 and extracellular volume fraction (ECV) maps acquired on a 1.5-T system in a patient with dilated cardiomyopathy (DCM). Significantly higher T1ρ values were observed in DCM patients (T1ρ  = 55.2 ± 2.7 ms) compared with heathy controls (T1ρ  = 51.5 ± 1.2 ms, P = 0.0024). T1ρ values correlated with ECV. [56] Reproduced with permission from van Oorschot et al.

DCM refers to a heterogeneous group of myocardial diseases with poor outcomes and with an estimated prevalence of 40 in 100 000 individuals [87, 88]. It is characterized by the presence of contractile dysfunction and left ventricular dilatation in the absence of severe coronary artery disease and abnormal loading conditions (hypertension, valvular heart disease) [89]. Assessment of mid-wall myocardial fibrosis with LGE imaging provides independent prognostic information in patients with non-ischemic DCM [87]. However, LGE fails to detect diffuse interstitial fibrosis, which is common pattern in patients with DCM [90]. Van Oorschot et al. [56] investigated the use of contrast-agent-free T1ρ mapping at 1.5-T to detect interstitial myocardial fibrosis in 20 patients suffering from end-stage DCM (Fig. 11). The authors found a significant elevation of T1ρ values in DCM patients (55.2 ± 2.7 ms) compared with healthy control subjects (51.5 ± 1.2 ms, P = 0.0024), as well as a significant correlation between ECV and T1ρ (Pearson r = 0.66). Native T1 was also elevated in DCM patients but no significant correlation was found between native T1 and T1ρ or ECV. Fibrosis fraction measured from histology in ex vivo human hearts also significantly correlated with T1ρ values. These promising results warrant longitudinal and large-scale multicenter studies to establish the diagnostic and prognostic power of contrast-agent-free myocardial T1ρ mapping in patients with DCM.

### Other cardiomyopathies

**Fig. 12.**
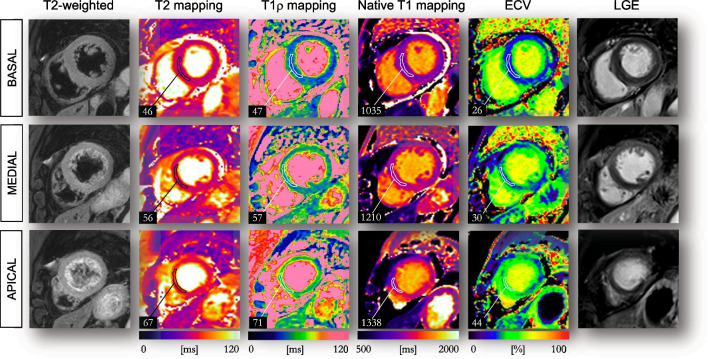
57-year-old female patient with findings consistent with stress-related Takotsubo cardiomyopathy. Myocardial T2 maps exhibit myocardial edema at mid and apical levels (T2 = 67 ms) with a clear T1ρ elevation at these locations (T1ρ  = 71 ms). Late gadolinium enhancement (LGE) images confirm the absence of myocardial necrosis. Images were collected on a 1.5-T system at Bordeaux University Hospital. *ECV* extracellular volume fraction

At present, myocardial T1ρ mapping has been limited to the above-mentioned myocardial disorders and clinical applications are still in their infancy. These preliminary findings warrant additional studies to investigate the clinical value and prognostic utility of T1ρ mapping in a host of other cardiomyopathies. Examples of subjects likely to benefit from such mapping technology are patients with Takotsubo cardiomyopathy (preliminary acquisitions in Fig. 12), muscular dystrophies, systemic cardiac disorders, or infiltrative and overload cardiomyopathies. The sensitivity and dynamic range of contrast-agent-free myocardial T1ρ mapping have also never been explored in patients with acute myocardial infarction and acute myocarditis. Its potential incremental value compared to established T2 mapping techniques remains to be investigated at this juncture. Considering that myocardial T1ρ mapping requires no pre- and post-contrast acquisitions, as for ECV mapping, it may therefore be easier to integrate it into routine clinical protocols and to compare its clinical value against established CMR techniques.

## Emerging technical developments and avenues toward clinical adoption

### High-resolution 3D T1ρ mapping and beyond

Although myocardial T1ρ mapping is still at a relatively early stage, promising 3D applications have already been proposed [52, 55, 91] and further developments in that direction are likely to emerge. Especially non-Cartesian myocardial T1ρ mapping remains to be implemented and thoroughly assessed. The so-called “free-running” framework [92] needs to be adapted to include spin-lock pulses for cardiac- and respiratory-resolved (5D) T1ρ mapping. On the motion side, more robust tools that could handle complex non-rigid deformations need to be rigorously developed and tested [42, 93]. CMR mapping techniques often sacrifice spatial resolution to yield quantitative information per pixel. This reduced spatial resolution could be a limiting factor of T1ρ mapping to detect myocardial damage, for example to discriminate subendocardial from subepicardial lesions, which is relevant for clinical diagnosis. Scar extent also being an important predictor of successful outcome after revascularization, resynchronization, ablation, or implantable cardioverter-defibrillator therapy [94, 95]. Therefore, further developments should aim at very high 3D spatial resolutions.

### Multi-parametric integration

Even though clinical studies have demonstrated the value of single mapping (T1, T2, T2*, ECV, …) techniques, and more recently T1ρ mapping, they are unable to provide a complete assessment of myocardial disease if they are applied and analyzed in silos. Multi-parametric mapping can provide a superior insight of myocardial tissue characteristics, more accurately identify myocardial injuries, while promoting knowledge and discovery. Magnetic resonance fingerprinting (MRF) and multitasking allow simultaneous measurement of multiple parameters (such as T1 and T2) in a single time-efficient scan and could thus provide a comprehensive assessment of the heart [96–98]. One of the advantages of MRF is that corrections for several confounding factors (see previous section) can also be included in the MRF dictionary (e.g., slice profile correction, T1ρ preparation pulse efficiency, B_1_^+^ field, etc.). Velasco et al. [99] recently made a first step in that direction by developing an MRF framework for simultaneous T1, T2, and T1ρ cardiac mapping in a single 16-s scan. The clinical integration and validation (including clinical trials) of the proposed technology are now awaited to establish its clinical utility.

### Artificial intelligence (AI) integration

The application of AI to CMR mapping is a very recent phenomenon. For myocardial T1ρ mapping, AI technologies could come in several flavors, including smarter acquisition schemes that lower scan times; advanced reconstructions that improve T1ρ map quality; automated segmentation that drives easier and more efficient analysis; and radiomics applications [100] to gain knowledge and make discoveries in a broad range of cardiac diseases. An example of such applications could be to train neural networks to perform myocardial T1ρ mapping with a reduced number of spin-lock pulses, as recently proposed by Guo et al. [101] for T1 mapping. This would be particularly interesting for 3D applications or to drastically shorten breath-holding time for 2D applications. AI could also be applied for T1ρ map quality control [102, 103], automated motion correction [104] and segmentation [105], and fully automated analysis and quantification [106]. Such applications would reduce the burden of manual analysis and operator variability and would represent a significant step towards clinical adoption.

### Standardization, reproducibility, availability, and adoption

The clinical studies discussed above provide exciting preliminary insights into contrast-agent-free quantification of myocardial injuries with myocardial T1ρ mapping. However, they also highlight important issues. First, and perhaps most importantly, widespread clinical adoption of myocardial T1ρ mapping is challenged by a lack of standardization and transferability (see for example the differing “normal” T1ρ values in Table 2) [58]. To this end, a standardized phantom should be employed as reference standard for true T1ρ relaxation and dispersion values. An agarose gel-based phantom (such as NIST or T1MES) or a phantom made of aqueous solutions of bovine serum albumin (or other proteins) could be used to evaluate the T1ρ relaxation characteristics for different systems, vendors, field strengths, and sequences, at different sites. We also encourage users to share the CMR protocols that specify the conditions in which T1ρ data are collected and the ways they are reconstructed. Furthermore, availability of myocardial T1ρ mapping is currently limited to a handful of specialized research centers and is not yet a clinical product. It is crucial that MR vendors provide T1ρ mapping sequences with flexible parameter setting options to fully explore the clinical potential of myocardial T1ρ mapping, to engage in more extensive exploratory efforts and to promote its widespread adoption.

Hopefully, more centers will soon get access to these new sequences on their clinical systems to initiate preclinical and clinical testing.

Finally, a sometimes-overlooked cornerstone of reproducibility is open access to data, reconstruction, and analysis code: this sharing enables others to check for bias against established mapping techniques. Efforts towards reproducibility have also been accelerated with the establishment of international networks such as the Quantitative Image Biomarker Alliance of the Radiological Society of North America (RSNA) and the Quantitative MR Study group of the International Society for Magnetic Resonance in Medicine (ISMRM). Both initiatives have put up much-needed roadmaps for the development of new quantitative imaging techniques that may help accelerate their uptake in routine clinical practice.

## Outlook and conclusions

The advances presented in this Review have shown the promising performance of myocardial T1ρ mapping for the quantification of myocardial injuries without the injection of contrast agents. The relatively early stage of the technique also leaves plentiful space for future work, both for technological and clinical research. On the technological side, myocardial T1ρ mapping can be further enhanced by encoding for 3D information; by integrating multiparametric technologies such as MRF; by exploiting AI-driven tools for faster, easier, and more efficient analysis with the potential of removing error-prone manual processes; and by exploring the parameter space of T1ρ mapping in terms of resolution, SNR, accuracy, and precision. On the clinical side, applications of myocardial T1ρ mapping are still in the early stages. However, the lack of clinical evidence from prospective and randomized trials should not be considered as a barrier to further technical developments and to the translation of already existing methods. Given the unique potential of the mapping technology, it is likely that there will soon be an multitude of clinical studies assessing the performance of T1ρ mapping in a wide range of clinical scenarios. And only when myocardial T1ρ mapping is fully tested, standardized, and released, will its true impact in healthcare become apparent.

## Data Availability

As part of the Open Science and reproducible research initiative, we provide phantom and in vivo T1ρ-weighted datasets at this repository: https://github.com/AurelienBustin/T1-rho-mapping. This repository also contains fitting codes as well as the T1ρ colormap used in this paper.

## Abbreviations

2D: 2-Dimensional
3D: 3-Dimensional
BOLD: Blood oxygen level-dependent
CEST: Chemical exchange saturation transfer
CMR: Cardiac magnetic resonance
DCM: Dilated cardiomyopathy
ECG: Electrocardiogram
ECV: Extracellular volume fraction
GBCAs: Gadolinium-based contrast agents
GRAPPA: Generalized autocalibrating partially parallel acquisitions
HCM: Hypertrophic cardiomyopathy
LGE: Late gadolinium enhancement
MRF: Magnetic resonance fingerprinting
RF: Radiofrequency
SAR: Specific absorption rate
SNR: Signal-to-noise ratio
T1ρ: T1-rho
